# Sheath-Assisted Redo Left Bundle Branch Area Pacing Without Additional Venous Puncture

**DOI:** 10.1016/j.jaccas.2026.109785

**Published:** 2026-08-10

**Authors:** Yohei Hiruma, Noriaki Moriyama, Hikaru Tanemura, Kunihiko Shimizu, Masato Murakami, Shigeru Saito

**Affiliations:** Department of Cardiology, Shonan Kamakura General Hospital, Kamakura, Japan

**Keywords:** cardiac pacemaker, complication, x-ray fluoroscopy

## Abstract

**Background:**

Left bundle branch area pacing (LBBAP) requires delivery-sheath support for implantation and revision. Redo procedures for lead-related complications may require additional venous puncture to regain access.

**Case Summary:**

A 79-year-old man underwent LBBAP for complete atrioventricular block. Four days later, abrupt threshold elevation and loss of capture occurred. Fluoroscopy showed complete interventricular septal perforation with lead-tip migration into the left ventricular cavity. Lead revision was performed using a sheath-assisted access-preserving technique without additional venous puncture. A modified 4-F short sheath was advanced over the existing lead, allowing guidewire insertion, sheath upsizing, and successful LBBAP lead reimplantation.

**Discussion:**

Delayed septal perforation is a rare but recognized LBBAP complication. This case demonstrates a simple technique using standard sheaths and guidewires that may reduce invasiveness during selected redo LBBAP procedures.

**Take-Home Message:**

Sheath-assisted access preservation can facilitate LBBAP lead revision without additional venous puncture.

## Visual Summary

**Figure:**
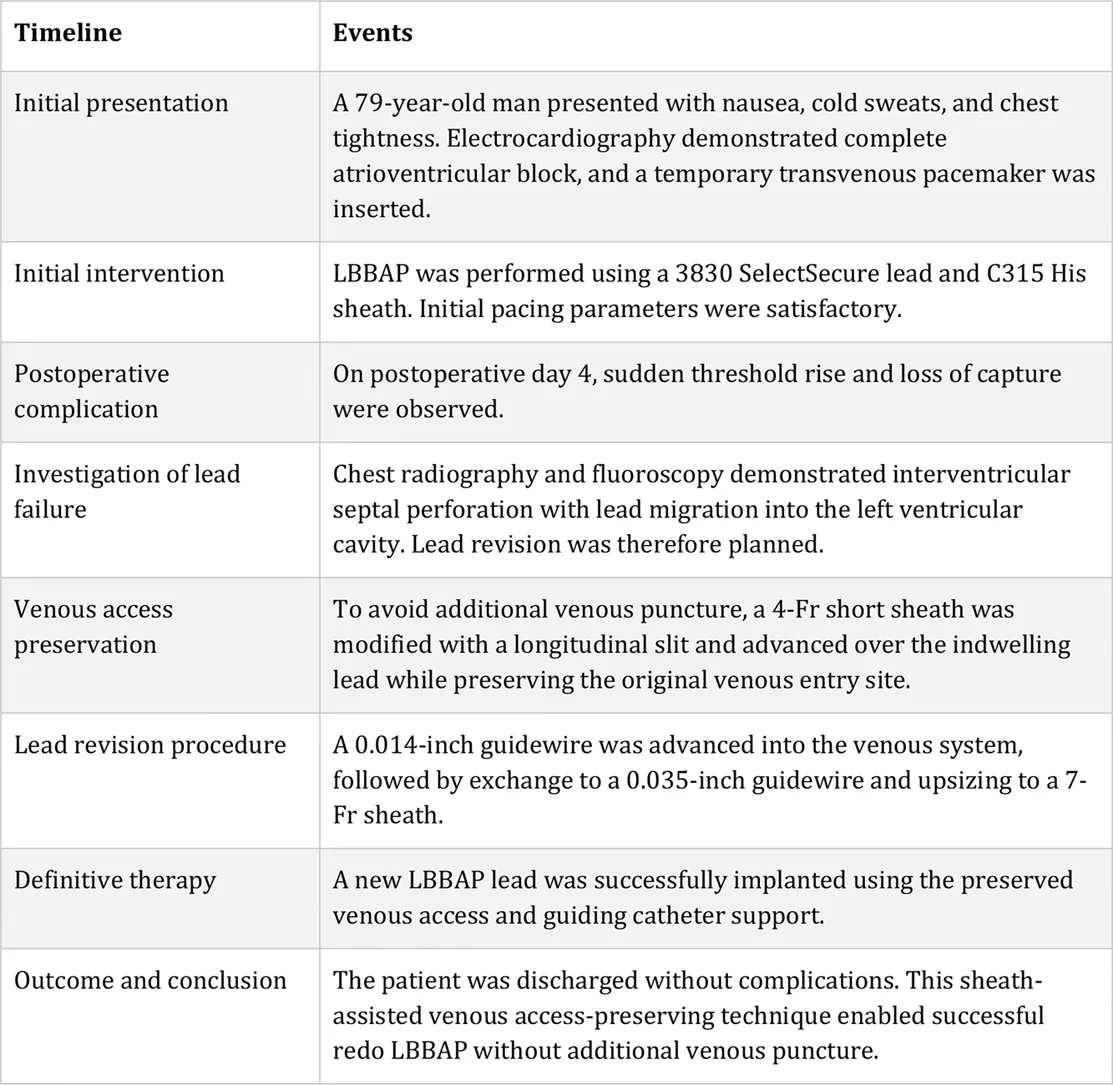

## History of Presentation

A 79-year-old man presented to the emergency department with nausea and cold sweats following morning urination. Initial examination and investigations were unremarkable, and he was discharged. However, later that evening, he returned to the emergency department with recurrence of symptoms during defecation, accompanied by chest tightness.

## Past Medical History

The patient had a history of myocardial infarction treated with percutaneous coronary intervention and dyslipidemia.

## Differential Diagnosis

The differential diagnosis of chest tightness included acute coronary syndrome and bradyarrhythmias, including complete atrioventricular block.

## Investigations

Electrocardiography revealed complete atrioventricular block with a ventricular escape rhythm at 53 beat/min (Figure 1). He was admitted under the cardiology service, and a temporary transvenous pacemaker was inserted. Coronary angiography showed no significant stenosis. On hospital day 4, a permanent pacemaker was implanted with a left bundle branch area pacing (LBBAP) approach using a 4.1-F lumenless 3,830-69 cm SelectSecure lead (Medtronic) through a C315 His sheath (Medtronic). Initial pacing parameters were satisfactory with a capture threshold of 0.5 V at 0.4 ms, an R-wave amplitude of 14.9 mV, and a lead impedance of 627 Ω. On postoperative day 4, device interrogation showed a sudden rise in ventricular pacing threshold (6.0 V at 1.0 ms, with noncapture), loss of injury current, and absence of effective pacing (Figure 2A). Chest radiography and fluoroscopy revealed migration of the ventricular lead tip beyond the septum into the left ventricular cavity, suggestive of complete interventricular septal (IVS) perforation (Figures 2B and 2C). Therefore, lead revision was planned immediately after diagnosis.t

**Figure 1:**
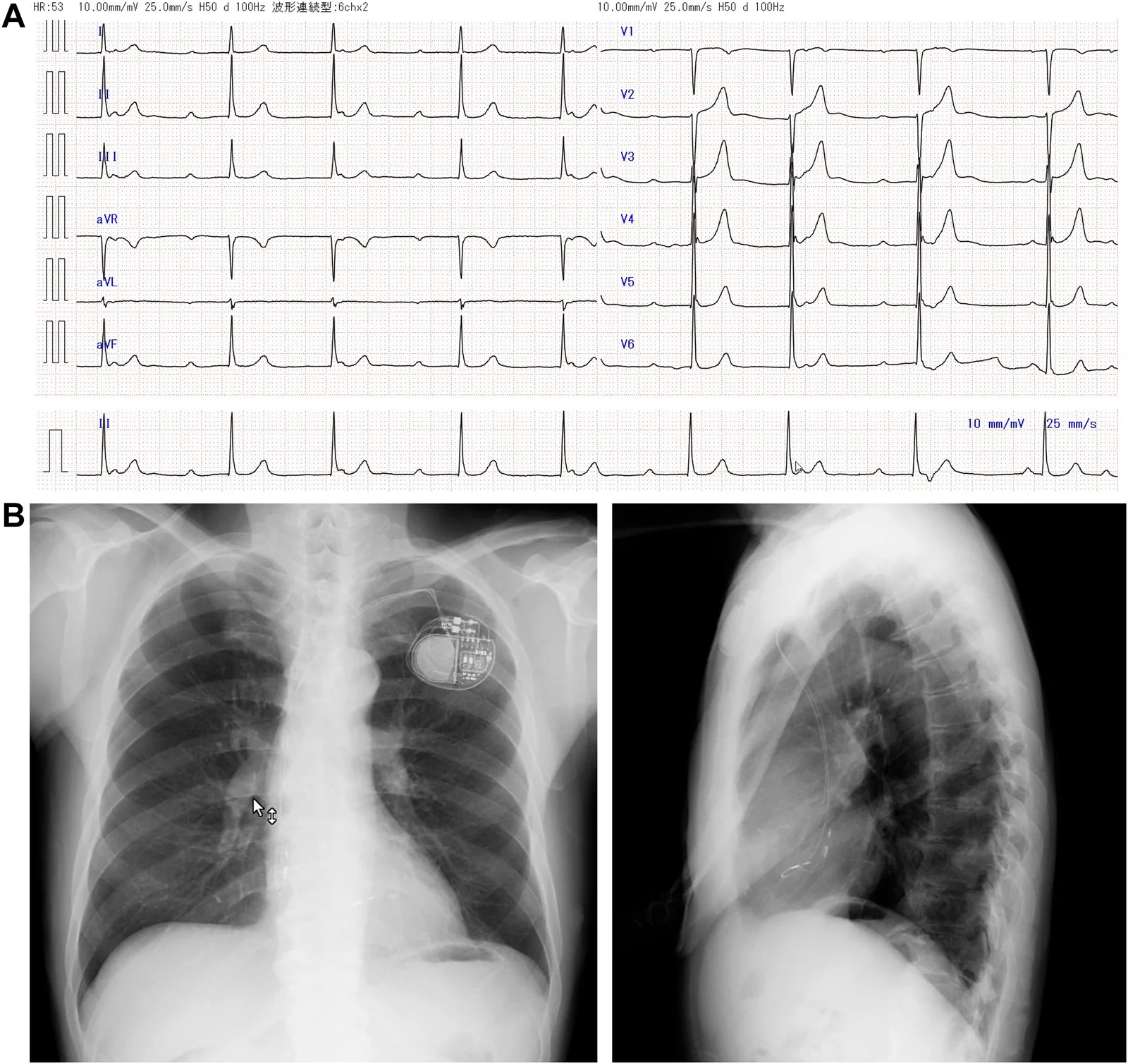
Initial Electrocardiography and Chest Radiography (A) Surface electrocardiographic morphology on presentation showing complete atrioventricular (AV) block. (B) Chest radiographs in posteroanterior projection (left) and lateral projection (right) showing the left bundle branch area pacing (LBBAP) lead and device position. aVF = augmented vector foot; aVL = augmented vector left; aVR = augmented vector right; PA = posteroanterior.

**Figure 2:**
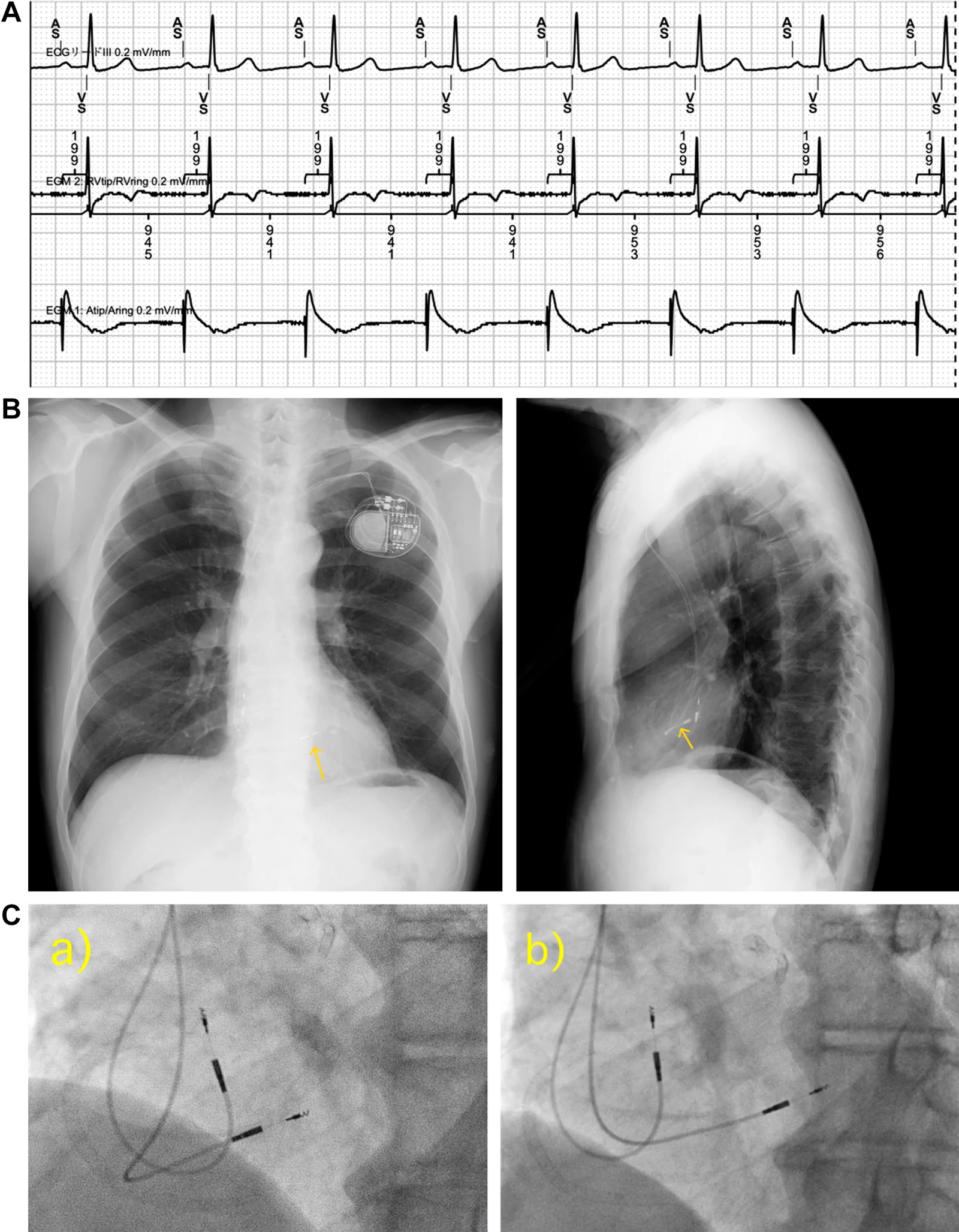
Diagnosis of Delayed Interventricular Septal Perforation (A) Intracardiac electrogram demonstrating disappearance of the injury current. (B) Chest radiographs in posteroanterior projection (left) and lateral projection (right) showing the left bundle branch area pacing (LBBAP) lead (yellow arrows) and device position. (C) Fluoroscopic images demonstrating ventricular lead migration in the left anterior oblique (LAO) 50° view. (a) At the initial implantation, the lead tip is located within the interventricular septum. (b) At the time of threshold rise, the lead tip has migrated beyond the septum into the left ventricular cavity. AS = atrial sense; EGM = electrogram; EOG = electrooculogram; PA = posteroanterior; VS = ventricular sense.

## Management

The lead revision procedure was performed under local anesthesia with additional sedation using propofol. The original incision was reopened, and the ventricular lead sleeve was exposed. To maintain the existing venous access and avoid a new puncture, we adopted a sheath-assisted technique. A single continuous longitudinal slit was created in the 4-F short sheath (TERUMO), extending approximately 5 cm proximally from its distal tip (Figure 3). The modified sheath was mounted onto the indwelling lead through the slit (Figure 4). Using a needle introducer, we confirmed that a 0.014-inch Cruise wire (Asahi Intecc) could pass through the access port of the sheath and reach its distal tip. The 4-F sheath was then advanced into the vessel by simultaneously pushing the lead; the sheath tip was successfully introduced into the venous lumen (Figure 5). After confirming backflow, correct intravascular positioning of the sheath was verified, and a 0.014-inch Cruise wire was successfully advanced into the inferior vena cava. Subsequently, the SelectSecure lead was removed while maintaining the position of the 4-F sheath. A 0.035-inch Radifocus wire (TERUMO) was inserted through the same access port, and the access was upsized to a 7-F sheath (Figure 6). A new SelectSecure lead was then delivered using a C315 His sheath, and successful LBBAP was achieved with a capture threshold of 0.25 V at 0.4 ms, an R-wave amplitude of 7.1 mV, and a lead impedance of 722 Ω (Figures 7 and 8). The incision was closed in 2 layers.

**Figure 3:**
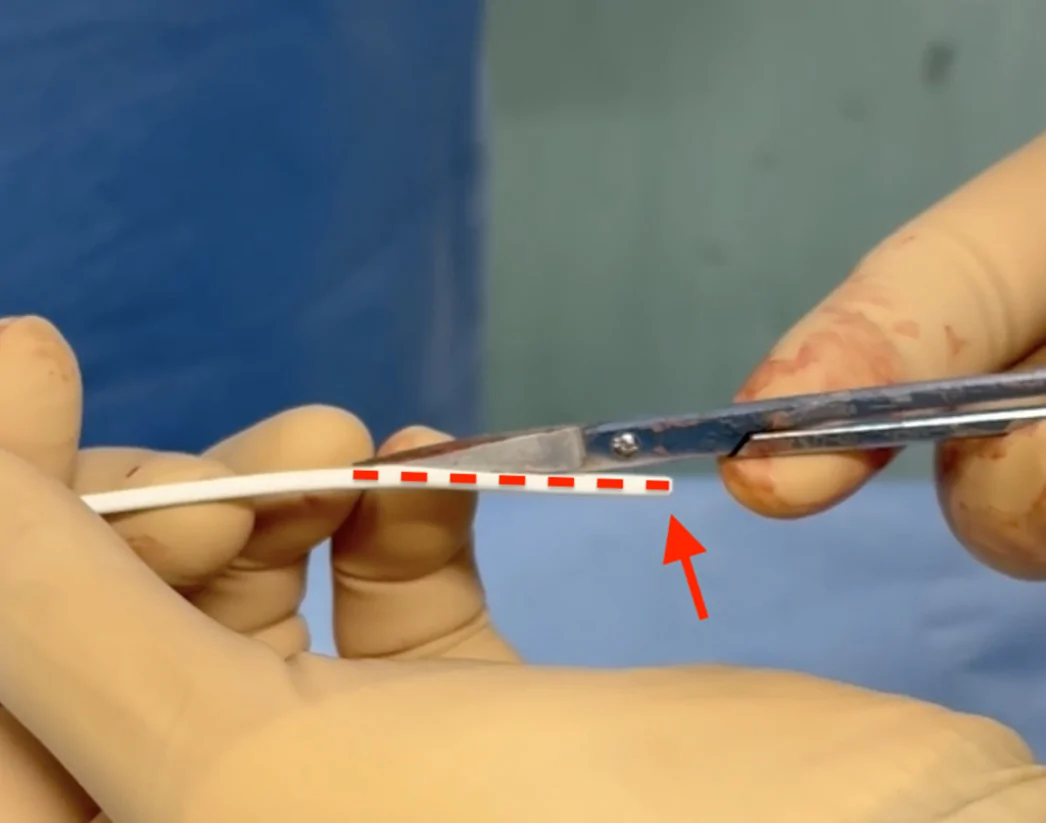
Modified 4-F Short Sheath A single continuous longitudinal incision was made along the sheath extending 5 cm proximally from its distal tip. The dashed red line indicates the longitudinal incision, and the red arrow indicates the distal tip.

**Figure 4:**
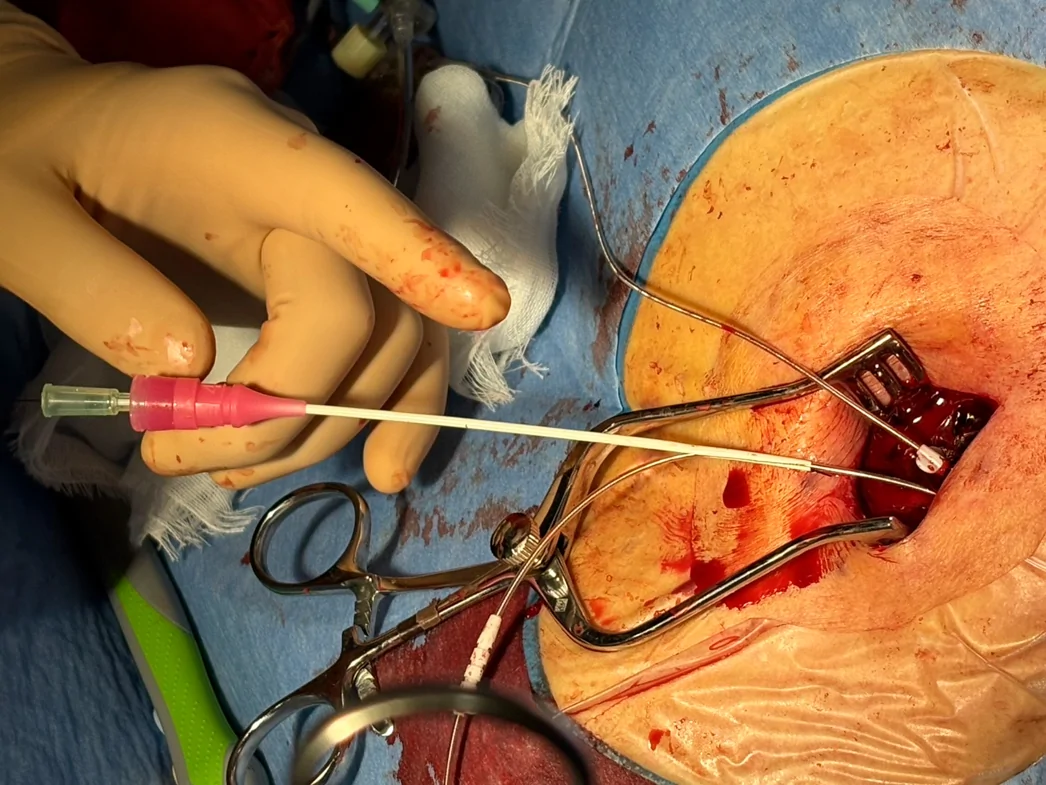
Mounting the Modified Sheath Onto the Indwelling Lead The modified sheath was mounted onto the indwelling lead through the longitudinal slit.

**Figure 5:**
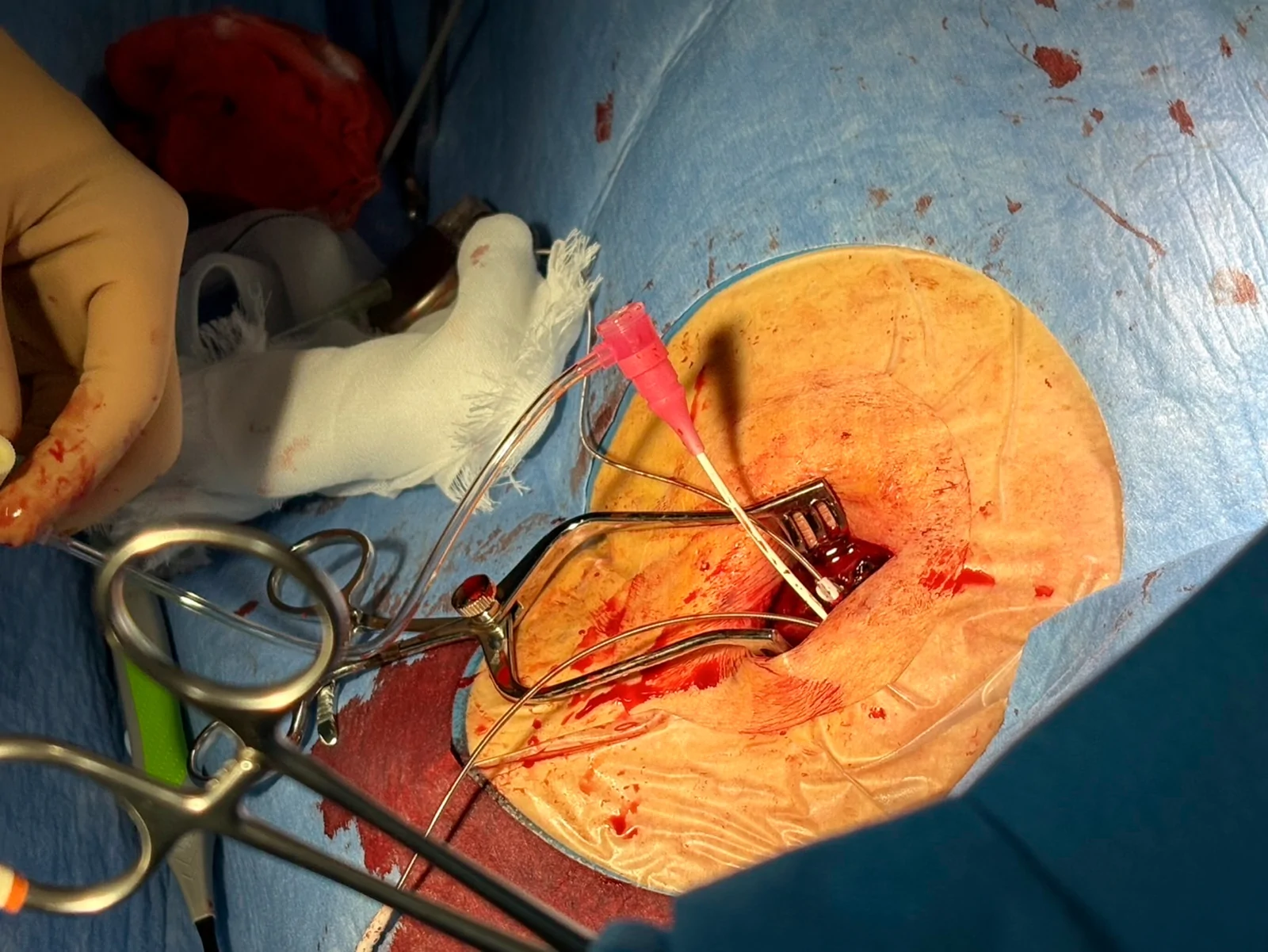
Sheath Advancement Into the Venous Lumen The modified 4-F sheath was advanced along the indwelling lead, and the sheath tip was introduced into the venous lumen.

**Figure 6:**
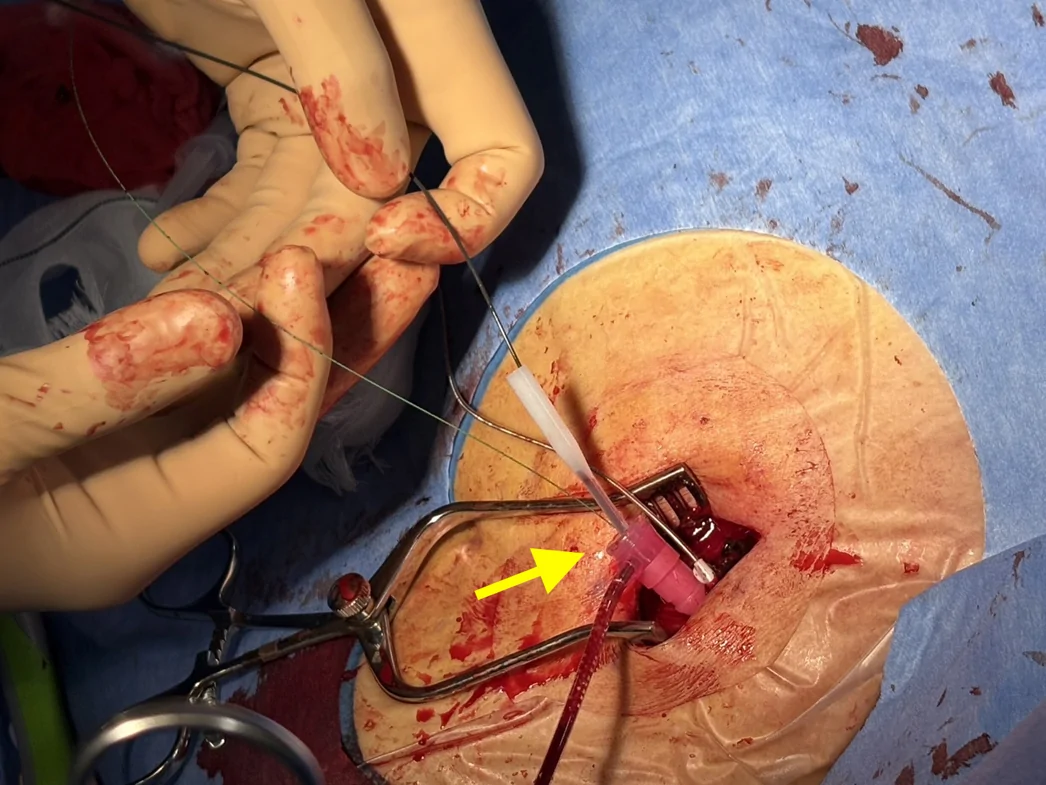
Guidewire Insertion Through the Access Port of the Modified 4-F Sheath A 0.014-inch Cruise wire and subsequently a 0.035-inch Radifocus wire were inserted through the access port of the modified 4-F sheath. The yellow arrow indicates the access port of the sheath.

**Figure 7:**
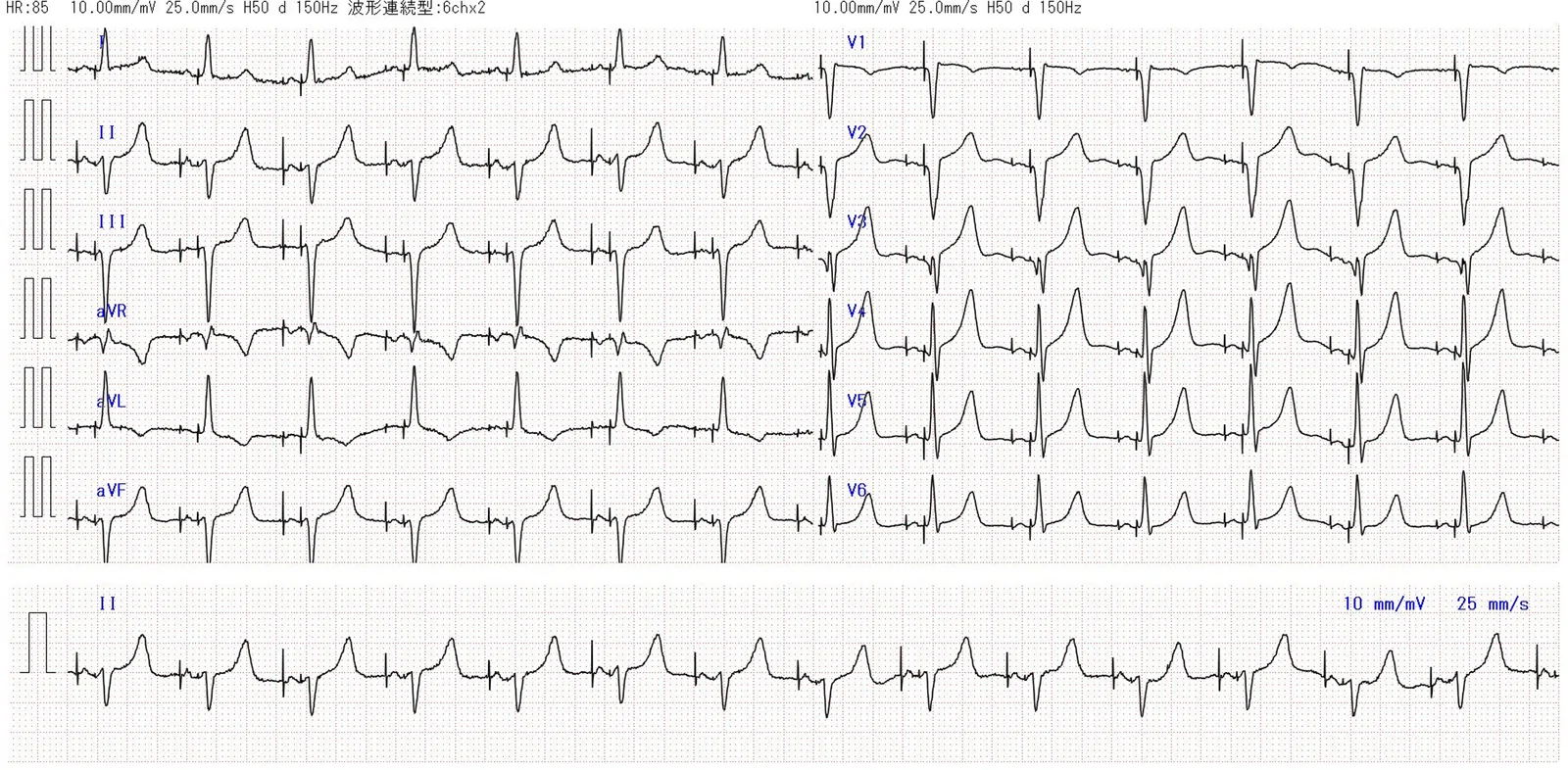
Electrocardiogram After Successful Left Bundle Branch Area Pacing Lead Reimplantation The 12-lead electrocardiogram after lead reimplantation demonstrates left bundle branch capture. aVF = augmented vector foot; aVL = augmented vector left; aVR = augmented vector right.

**Figure 8:**
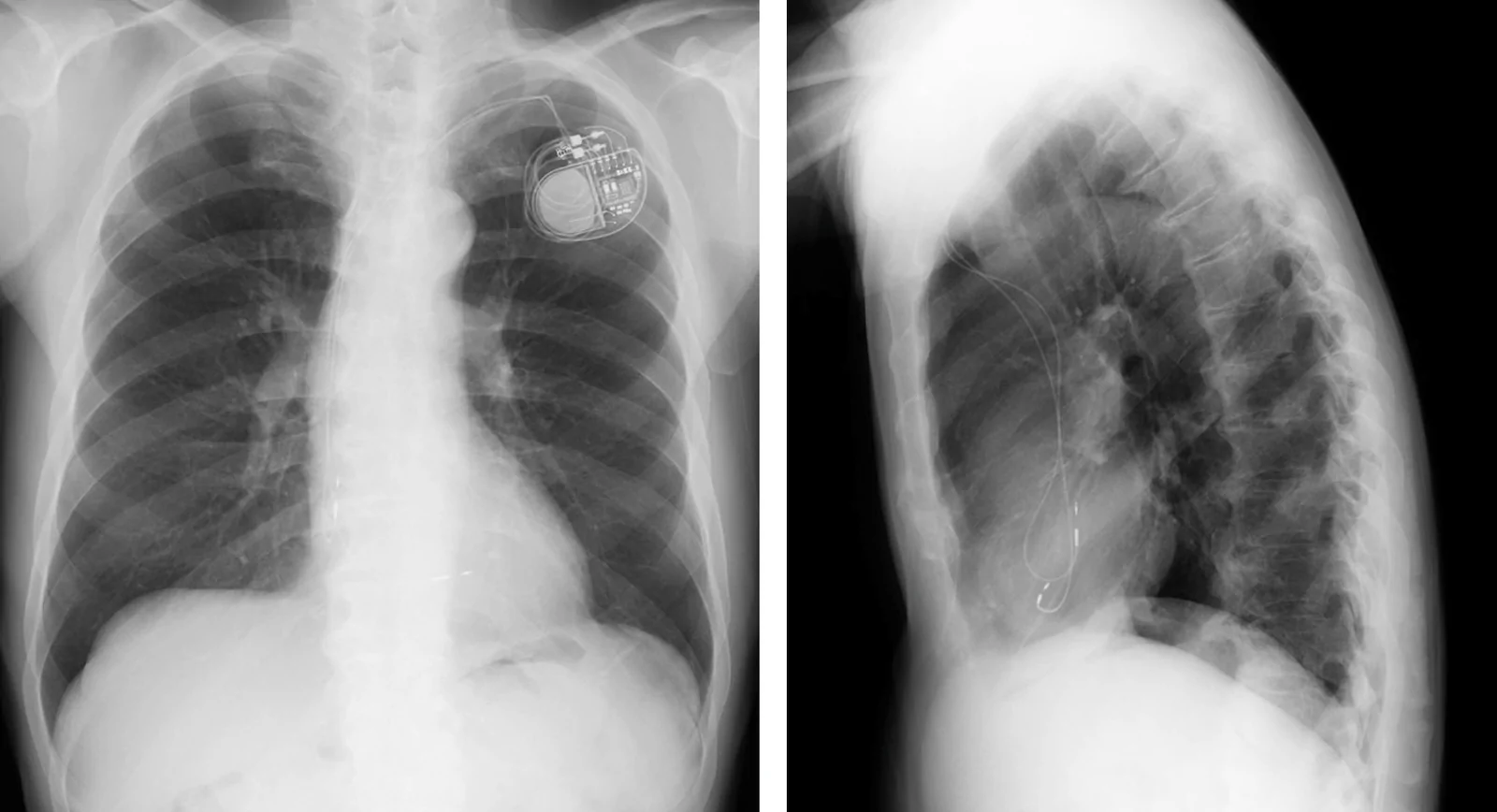
Postprocedural Chest Radiographs After Pacemaker Implantation Postprocedural chest radiographs in the posteroanterior (left) and lateral (right) views demonstrate the final positions of the pacemaker leads after successful reimplantation of the left bundle branch area pacing lead.

## Outcome and Follow-Up

The patient remained hemodynamically stable and was discharged on hospital day 12 with no further complications.

## Discussion

Conduction system pacing, particularly LBBAP, has gained traction as a physiological alternative to right ventricular pacing, aiming to preserve ventricular synchrony and reduce the risk of pacing-induced cardiomyopathy. Compared with His bundle pacing, LBBAP provides more stable pacing thresholds and lead fixation, and is therefore increasingly used in patients requiring high-burden ventricular pacing.^1^ Unlike conventional right ventricular lead implantation, LBBAP requires guiding catheter support to achieve accurate septal engagement and fixation.^2^

LBBAP implantation is generally safe; however, several procedure-specific complications have been reported. Acute IVS perforation has been reported in a small proportion of cases during implantation, whereas subacute or delayed perforation remains uncommon and is poorly characterized in the literature.^3^^,^^4^ Delayed septal perforation has been reported in approximately 0.1%-0.3% of cases.^5^^,^^6^ With the expanding adoption of LBBAP, the number of lead-related complications, including dislodgement or septal perforation, is expected to increase.

When redo LBBAP is required because of lead dislodgement or septal perforation, simple repositioning is often insufficient because precise septal re-engagement depends on guiding catheter support. Consequently, revision procedures frequently require a new venous puncture to reintroduce the guiding catheter. However, additional venous puncture during lead revision may increase procedural invasiveness, bleeding risk, and the likelihood of venous stenosis or occlusion, particularly in elderly patients or those requiring lifelong device therapy. Preservation of the original venous access may therefore offer important procedural and anatomical advantages.

The key novelty of this case lies in a venous access–preserving strategy that enables LBBAP lead revision without additional venous puncture. By utilizing a trimmed small-diameter sheath advanced over the existing lead through a longitudinal slit, we recreated a stable intravascular pathway that allowed stepwise guidewire insertion and sheath upsizing while maintaining the original venous entry site. This approach is particularly relevant in revision scenarios where guiding catheter support is essential for successful reimplantation. As LBBAP implantation remains dependent on delivery-sheath–guided techniques, revision strategies that avoid repeat venous puncture may become increasingly important. The method described here requires no specialized equipment beyond standard short sheaths and guidewires, making it potentially reproducible in a wide range of institutions.

Although this technique was used for an acute revision of a small-caliber lumenless LBBAP lead in the present case, we have also successfully applied a similar sheath-assisted approach to an 8.6-F stylet-driven implantable cardioverter defibrillator lead 2 months after implantation, using an 8-F sheath. In both procedures, selecting a sheath approximately 1-F smaller than the sheath originally used for lead implantation appeared to be an important technical consideration. This experience suggests that the technique may be feasible beyond the acute phase and with selected larger-caliber stylet-driven leads. However, because our experience remains limited, its generalizability to chronically implanted leads with fibrotic adhesions and to other lead types, including stylet-driven LBBAP leads, requires further evaluation.

## Conclusions

Lead dislodgement and IVS perforation are rare but important complications of LBBAP. Our case emphasizes a sheath-assisted technique that enabled successful lead revision without additional venous access. Awareness of such troubleshooting strategies may facilitate safer and more efficient management of similar complications in the growing field of conduction system pacing.

**Take-Home Messages**

• Delayed interventricular septal perforation is a rare but clinically significant complication of left bundle branch area pacing that may present with sudden threshold rise and loss of capture.
• Redo left bundle branch area pacing procedures typically require new venous access; however, a sheath-assisted venous access-preserving technique can enable safe and effective lead revision without additional venous puncture.

## Funding Support and Author Disclosures

The authors have reported that they have no relationships relevant to the contents of this paper to disclose.

**Visual Summary:** Timeline of Sheath-Assisted Left Bundle Branch Area Pacing Lead Revision

Timeline	Events
Initial presentation	A 79-y-old man presented with nausea, cold sweats, and chest tightness. Electrocardiography demonstrated complete atrioventricular block, and a temporary transvenous pacemaker was inserted.
Initial intervention	LBBAP was performed using a 3,830 SelectSecure lead and C315 His sheath. Initial pacing parameters were satisfactory.
Postoperative complication	On postoperative d 4, sudden threshold rise and loss of capture were observed.
Investigation of lead failure	Chest radiography and fluoroscopy demonstrated interventricular septal perforation with lead migration into the left ventricular cavity. Lead revision was therefore planned.
Venous access preservation	To avoid additional venous puncture, a 4-F short sheath was modified with a longitudinal slit and advanced over the indwelling lead while preserving the original venous entry site.
Lead revision procedure	A 0.014-inch guidewire was advanced into the venous system, followed by exchange to a 0.035-inch guidewire and upsizing to a 7-F sheath.
Definitive therapy	A new LBBAP lead was successfully implanted using the preserved venous access and guiding catheter support.
Outcome and conclusion	The patient was discharged without complications. This sheath-assisted venous access-preserving technique enabled successful redo LBBAP without additional venous puncture.

**Equipment List:** Equipment List Used in the Procedure

Sheath-Assisted Venous Access Preservation • 4-F short sheath (TERUMO) • Needle introducer • 0.014-inch Cruise guidewire (Asahi Intecc) • 0.035-inch Radifocus guidewire (TERUMO) • 7-F short sheath (TERUMO)
Left Bundle Branch Area Pacing Lead Reimplantation • C315 His delivery sheath (Medtronic) • 3,830-69 cm SelectSecure 4.1-F lumenless lead (Medtronic)

## References

[1] Burri H., Jastrzebski M., Cano Ó. (2023). EHRA clinical consensus statement on conduction system pacing implantation: endorsed by the Asia Pacific Heart Rhythm Society (APHRS), Canadian Heart Rhythm Society (CHRS), and Latin American Heart Rhythm Society (LAHRS). Europace.

[2] Cano Ó., Vijayaraman P. (2021). Left bundle branch area pacing: implant technique, definitions, outcomes, and complications. Curr Cardiol Rep.

[3] Stempfel S., Tolppanen H., Valiton V., Burri H. (2025). Complications with left bundle branch area pacing: the flip side of the coin. Heart Int.

[4] Ravi V., Larsen T., Ooms S. (2020). Late--onset interventricular septal perforation from left bundle branch pacing. HeartRhythm Case Rep.

[5] Jastrzębski M., Kiełbasa G., Cano O. (2022). Left bundle branch area pacing outcomes: the multicentre European MELOS Study. Eur Heart J.

[6] Chen X., Wei L., Bai J. (2021). Procedure—related complications of left bundle branch pacing: a single-center experience. Front Cardiovasc Med.

